# Ordering patterns for laboratory and radiology tests by students from different undergraduate medical curricula

**DOI:** 10.1186/1472-6920-13-109

**Published:** 2013-08-14

**Authors:** Sigrid Harendza, Lonneke Alofs, Jorike Huiskes, Marjo Wijnen-Meijer

**Affiliations:** 1 Department of Internal Medicine, University Medical Centre Hamburg-Eppendorf, Martinistr. 52, 20246 Hamburg, Germany; 2 Center for Research and Development of Education, UMC Utrecht, P.O. Box 85500, Utrecht, GA 3508 The Netherlands

**Keywords:** Laboratory test, Radiology test, Requesting pattern, Clinical reasoning, Competence, Differential diagnosis

## Abstract

**Background:**

The overuse of laboratory tests and radiology imaging and their possible hazards to patients and the health care system is observed with growing concern in the medical community. With this study the authors wished to determine whether ordering patterns for laboratory and radiology tests by medical students close to their graduation are related to undergraduate training.

**Methods:**

We developed an assessment for near graduates in the setting of a resident’s daily routine including a consultation hour with five simulated patients, three hours for patient work up with simulated distracting tasks, and thirty minutes for reporting of patient management to a supervisor. In 2011, 60 students participated in this assessment: 30 from a vertically integrated (VI) curriculum (Utrecht, The Netherlands) and 30 from a traditional, non-VI curriculum (Hamburg, Germany). We assessed and compared the number of laboratory and radiology requests and correlated the results with the scores participants received from their supervisors for the facet of competence “scientifically and empirically grounded method of working”.

**Results:**

Students from a VI curriculum used significantly (p < .01) less total laboratory requests (N = 283 versus N = 466) which correlated with their scores for a “scientifically and empirically grounded method of working” (Pearson’s r = .572). A significantly (p < .01) higher number of radiology imaging was ordered with a large effect size (V = .618) by near graduates from a non-VI curriculum (N = 156 versus N = 97) even when this was not supporting the diagnostic process.

**Conclusion:**

The focused ordering patterns from VI students might be a result of their early exposure to the clinical environment and a different approach to clinical decision making during their undergraduate education which further studies should address in greater detail.

## Background

The overuse of laboratory tests and computer tomography (CT) remains a problem amongst physicians [1,2]. Two thirds of common laboratory investigations ordered during the hospitalisation of patients have no influence on management decisions [3]. Reasons for excessive ordering of tests by doctors include defensive behaviour and uncertainty, lack of experience, inadequate educational feedback, and clinicians’ unawareness about costs [4,5]. A study from Israel found that almost 70% of participating medical students did not receive any information about costs of medical tests during their undergraduate training [6]. A Belgian study revealed that physicians in an emergency department had limited knowledge of costs and radiation doses of the investigations they prescribed every day [7]. However, a combination of administrative changes and physician education initiatives can influence physicians’ test-ordering behaviour [8]. Ordering guidelines or clinical guidelines can also alter the ordering patterns of physicians and improve the appropriateness of laboratory tests and chest radiographs [9,10].

A study from the early 90s of the last century demonstrated that the medical history led to the final diagnosis in 76% of patients from an outpatient clinic while laboratory investigations did so in only 11% [11]. Additionally, medical students had difficulties selecting and justifying laboratory tests that would further narrow diagnostic possibilities and link the final diagnosis to the specific aspects of the history, physical examination and results of laboratory tests [12]. It has been assumed that making a diagnosis has shifted from the history and the physical examination to results of laboratory tests because faculty members are more often away from the bedside [13]. Therefore, case-based teaching of clinical reasoning has been proposed as a useful method to study the diagnostic process and to learn about the complex trade-offs between the benefits and risks of diagnostic tests [14].

Many medical schools have changed their undergraduate curricula to vertically integrated (VI) programmes to provide early clinical experiences and progressively increasing clinical responsibility [15-18]. This could ease the transition from student to doctor including the acquisition of clinical reasoning strategies and responsible ordering patterns for laboratory tests and radiology imaging. Non-VI curricula equip students with many basic science facts and clinical details which they might try to apply in patient care [19]. VI curricula contain fewer details of basic science and clinical knowledge [20] which, on the other hand, might force students to apply different strategies of approaching a patient problem. Therefore, the research question of our study was: Do near graduates from a VI curriculum display a different ordering pattern for laboratory and radiology tests than near graduates from a non-VI curriculum and are these patterns associated with their respective competence score for a “scientifically and empirically grounded method of working”?

## Methods

### Design and sample

We developed and conducted a performance assessment for medical students near graduation named UHTRUST (Utrecht Hamburg Trainee Responsibility for Unfamiliar Situations Test) which is described and provided with a validity argument elsewhere [21,22]. In brief, voluntary participants were put in the position of beginning residents on a very busy day. First, each candidate had to see five standardized patients in an outpatient clinic (1 hour). Secondly, candidates were supposed to request additional information, e.g. laboratory tests, and to design examination or treatment schemes while confronted with seven realistic distracting tasks, e.g. questions from nurses (3 hours), and thirdly, candidates reported their differential diagnoses and management proposals for each patient to their supervising physicians (30 minutes).

Five patients presented with their respective problems: Case 1: 5-year-old girl – case presented by her mother – with weariness and abdominal pain (diagnosis: coeliac disease), case 2: 53-year-old man with haemoptysis and fatigue (diagnosis: Wegener’s granulomatosis), case 3: 58-year-old woman with abdominal pain (diagnosis: perforated sigmoid diverticulitis), case 4: 65-year-old woman – accompanied by her husband – with difficulties to speak and to swallow (diagnosis: myasthenia gravis), case 5: 36-year-old man with rheumatoid arthritis and fever (diagnosis: varicella zoster infection).

All candidates were assessed on different facets of competence (FOCs) [23] by physicians, nurses and standardized patients. Sixty medical students near graduation participated during the assessment days in July and August 2011. At Utrecht University Hospital, a total of 30 near graduates (23 from Utrecht, 7 from Groningen) and at Hamburg University Hospital, 30 near graduates from Hamburg attended the assessment.

### Instruments and data collection

Participants’ performance was assessed regarding several FOCs with a set of different scoring forms described in further detail elsewhere [21]. The relevant instruments used for this particular study were the supervisors’ ratings of the candidates regarding the FOC “scientifically and empirically grounded method of working” on a 3-point Likert scale of 1 (weak) to 3 (good). This FOC is described as follows: “The physician uses evidence-based procedures whenever possible and relies on scientific knowledge. He searches actively and purposefully for evidence and consults high quality resources. He uses his scientific knowledge critically and carefully in his work [23]”.

Laboratory and radiology requests were counted for each candidate and country according to the following instructions. Participants from both countries used the typical ordering system of their training hospital. In Utrecht, candidates received regular, hospital adapted application forms for laboratory and radiology requests where they could mark the investigations they requested. In Hamburg, candidates were supplied with blank ordering forms where they could write in key words all investigations they wanted to order. Laboratory requests were counted in the following categories: “haematology”, “clinical chemistry”, “blood gas analysis”, “clotting tests”, “immunology”, and “others”. When Dutch participants, for example, asked for sodium, potassium and haemoglobin, they received one count for “clinical chemistry” and one count for “haematology”. When German candidates, for example, asked for “blood count” the way it is usually done in their setting, they received one count for “haematology” even though the “blood count” includes haemoglobin, leukocytes, platelets, MCH and MCV which can all also be ordered individually. Radiology requests were counted in the categories “X-rays” and “CT-scans and other imaging”.

### Statistical analysis

We assessed differences between the candidate groups from both countries by χ^2^-tests regarding the number of laboratory or radiology requests within the different categories and in total for laboratory or radiology requests, respectively with significance levels of p < .05. Effect sizes (Cramer’s V) are also provided with values of .1 indicating small and values of > .5 indicating large effects. We calculated Pearson correlation coefficients (r) between the mean score of two physicians for the FOC “scientifically and empirically grounded method of working” and the number of total laboratory and radiology requests.

### Ethical approval

Ethical approval for the Dutch part of the study was obtained from the NVMO Ethical Review Board. For the German part, ethical approval was obtained from the State of Hamburg Physicians’ Ethics Board. All subjects provided written informed consent to participate in the study.

## Results

### Laboratory requests

German candidates requested a significantly higher number of total laboratory tests (p < .01) with a large effect size (V = .754). The number of laboratory tests ordered individually for each of the five patients was also significantly higher (p < .05 and < .01, respectively) for German candidates in all cases (Table 1). The number of total laboratory tests showed a significant correlation with the achieved score in the FOC “scientifically and empirically grounded method of working” for Dutch (Pearson’s r = .572) but not for German participants. The most prominent difference in laboratory test ordering patterns by Dutch and German candidates was found in the category “clotting test” (Table 1). For all individual patients and in total, German candidates ordered significantly more clotting tests compared with Dutch candidates (p < .05 and .01 respectively).

**Table 1:** Laboratory and radiology requests

**Patient case**	**Total laboratory request (n)**	**Requests for clotting tests (n)**	**Total radiology requests (n)**	**Requests for CT scans (n)**
	**NL / G**	**NL / G**	**NL / G**	**NL / G**
**1**	47 / 85**	0 / 7**	7 / 20**	0 / 20**
**2**	69 / 116**	4 / 16**	28 / 38	4 / 11*
**3**	54 / 83**	1 / 17**	36 / 46	24 / 25
**4**	50 / 84*	0 / 8**	7 / 19*	3 / 11*
**5**	63 / 98**	4 / 12*	19 / 33**	0 / 5*
**Total**	283 / 466**	9 / 60**	97 / 156**	31 /72**

*n*  number, *NL*  The Netherlands, *G*  Germany, * p < .05; ** p < .01.

### Radiology requests

A significantly higher number of radiology requests was made by German candidates (Table 1) compared with Dutch candidates (p < .01) with a large effect size (V = .618). No significant correlation was found for the total number of radiology requests and the score for the FOC “scientifically and empirically grounded method of working” in either country. Furthermore, German candidates requested more than twice as many CT-scans and other imaging compared with Dutch candidates (p < .01) (Table 1). The most significant difference (p < .001) was found for case 1 (5-year-old girl with weariness and abdominal pain) whereas no significant difference could be found for case 3 (58-year-old woman with perforated sigmoid diverticulitis).

## Discussion

Near graduates from a VI curriculum (The Netherlands) ordered significantly less laboratory tests for the same patients they had to manage in this assessment compared with near graduates from a non-VI curriculum (Germany). A high number of laboratory requests was only associated with high scores for the FOC “scientifically and empirically grounded method of working” in participants from a VI curriculum. These findings support our hypothesis that students from a VI curriculum might apply different ways in approaching and managing patients. Early clinical experiences and involvement with patient care might shape students’ diagnostic performances [24]. They use evidence-based laboratory test approaches such as testing only where it seems appropriate, knowing about the nature and quality of evidence required for the clinical utility of a test, and learning how a test result impacts on clinical actions [25]. Supporting this hypothesis further, it has been demonstrated in a Swedish study that fifth year medical students from a non-VI curriculum requested a significantly greater number of laboratory tests for a given number of primary health care cases than physicians undergoing postgraduate training in general medicine which was interpreted to reflect differences in clinical experience [19].

It has been shown for certain blood tests, e.g. uric acid, that physicians order these “out of habit” [26] which might have been the case for clotting tests by near graduates form the non-VI curriculum in Germany. For all patients in our setting, clotting tests were within normal range and according to the design of the patient cases neither a normal nor a pathological clotting test would have provided helpful information to support or to exclude a potential differential diagnosis. The fact, that the number of laboratory tests decreases with physicians’ cumulative experience in caring for a patient’s primary diagnosis [27] supports early clinical training for medical students as provided in VI curricula. Furthermore, clinical experience and clinical reasoning strategies facilitate estimation of the pre-test probability for a disease leading to the correct selection of laboratory tests [28]. To further improve and manage demands for laboratory tests a computerised laboratory management and reimbursement system based on diagnosis-treatment combinations seems to be promising [29]. This could be a useful tool in undergraduate and postgraduate medical education to support patient management strategies and to lower unnecessary laboratory costs.

Radiology tests were also ordered to a significantly greater extent by near graduates from a non-VI curriculum. This difference was most prominent for CT scans where only one of the five patients in our study (case 3) would have needed a CT scan to confirm her diagnosis which was correctly ordered by most of the VI curriculum near graduates. A growing overutilization of radiology procedures at times when they will not improve diagnostic processes or patient outcomes has been noted in general [30]. Its potential hazard to patients by overexposing them to unnecessary radiation doses has been described [30]. A lack of certainty, confidence, or experience in the diagnosis has been identified as potential reasons for an overuse of imaging procedures [31]. Very worrisome is the fact that two thirds of participants from the non-VI curriculum in our study wanted to perform a CT scan in a 5-year-old child where this investigation would not have been necessary to work out the correct diagnosis. There is growing concern in the medical community about the increasing number of unnecessary CT scans, especially for children [32]. The use of CT scans in children with a cumulative dose about 50 to 60 mGy has recently been shown to be associated with an increased risk of leukaemia and brain cancer, respectively [33]. Physicians’ unawareness of such radiation risks has been of major concern in recent years [34]. As for laboratory tests, a computerized radiology order entry system with decision support based on clinical reasoning strategies and utility scores on the indications was demonstrated to have an important impact on physicians’ ordering practices [35]. Early clinical experiences and increasing responsibilities with feedback as provided in VI curricula in medical undergraduate education might have a similar effect.

We newly developed and validated [22] this prototypic assessment for medical graduates in a simulated realistic work situation. Furthermore, voluntary participants were not under the pressure of a real assessment situation and could behave “normally”. On the other hand, because participation was voluntary only very motivated students might have participated which could challenge the results. Also, participants were not only near graduates from different types of medical curricula but also from different countries which might reflect certain cultural differences. Participants also used different techniques for their laboratory and radiology requests, simulating the system they were accustomed to in either country for best realistic performance. Furthermore, a more cost-conscience organizational culture in one country or possible variance in local strategies regarding laboratory ordering could account for portions of the variance observed. Even taking such biases into account, our results appear considerably unambiguous and apparent.

## Conclusions

Our study shows that ordering patterns for laboratory tests correlate highly with the score for a “scientifically and empirically grounded method of working” in near graduates from a VI curriculum. These near graduates also order significantly less radiology tests than near graduates from a non-VI curriculum and use CT scans predominantly in situations when they are absolutely required in the diagnostic process. We conclude that students from a VI curriculum are trained to acquire laboratory and radiology test in a much more patient and diagnosis oriented way which might be a result of their early exposure to the clinical environment and their increasing clinical responsibility during undergraduate medical education. This should be addressed in further studies about clinical decision making.

## Abbreviations

CT: Computer tomography; FOC: Facet of competence; MCH: Mean cell haemoglobin; MCV: Mean corpuscular volume; VI: Vertically integrated.

## Competing interests

The authors declare that they have no competing interests.

## Authors’ contributions

All authors have contributed sufficiently to the project to be included as authors: MWM and SH designed the study, JH acquired the data, LA performed the statistical analysis. SH and MWM drafted the manuscript. All authors read and approved the final manuscript.

## Pre-publication history

The pre-publication history for this paper can be accessed here:

http://www.biomedcentral.com/1472-6920/13/109/prepub
